# The Association Between Recanalization, Collateral Flow, and Reperfusion in Acute Stroke Patients: A Dynamic Susceptibility Contrast MRI Study

**DOI:** 10.3389/fneur.2019.01147

**Published:** 2019-10-25

**Authors:** Kersten Villringer, Sascha Zimny, Ivana Galinovic, Christian H. Nolte, Jochen B. Fiebach, Ahmed A. Khalil

**Affiliations:** ^1^Center for Stroke Research Berlin, Charité-Universitätsmedizin Berlin, Berlin, Germany; ^2^Department of Neurology, Ev.-Luth. Diakonissenanstalt zu Flensburg, Flensburg, Germany; ^3^Department of Neurology, Charité-Universitätsmedizin Berlin, Berlin, Germany; ^4^Department of Neurology, Max Planck Institute for Human Cognitive and Brain Sciences, Leipzig, Germany; ^5^Mind, Brain, Body Institute, Berlin School of Mind and Brain, Humboldt-Universität Berlin, Berlin, Germany; ^6^Berlin Institute of Health, Berlin, Germany

**Keywords:** reperfusion, collateral flow, recanalization, stroke, MRI

## Abstract

**Background:** Collateral circulation in ischemic stroke patients plays an important role in infarct evolution und assessing patients' eligibility for endovascular treatment. By means of dynamic susceptibility contrast MRI, we aimed to investigate the effects of reperfusion, recanalization, and collateral flow on clinical and imaging outcomes after stroke.

**Methods:** Retrospective analysis of 184 patients enrolled into the prospective observational 1000Plus study (clinicaltrials.org NCT00715533). Inclusion criteria were vessel occlusion on baseline MR-angiography, imaging within 24 h after stroke onset and follow-up perfusion imaging. Baseline Higashida score using subtracted dynamic MR perfusion source images was used to quantify collateral flow. The influence of these variables, and their interaction with vessel recanalization, on clinical and imaging outcomes was assessed using robust linear regression.

**Results:** Ninety-eight patients (53.3%) showed vessel recanalization. Higashida score (*p* = 0.002), and recanalization (*p* = 0.0004) were independently associated with reperfusion. However, we found no evidence that the association between Higashida score and reperfusion relied on recanalization status (*p* = 0.2). NIHSS on admission (*p* < 0.0001) and recanalization (*p* = 0.001) were independently associated with long-term outcome at 3 months, however, Higashida score (*p* = 0.228) was not.

**Conclusion:** Higashida score and recanalization were independently associated with reperfusion, but the association between recanalization and reperfusion was similar regardless of collateral flow quality. Recanalization was associated with long-term outcome. DSC-based measures of collateral flow were not associated with long-term outcome, possibly due to the complex dynamic nature of collateral recruitment, timing of imaging and the employed post-processing.

## Introduction

In stroke patients with occlusions of large, proximal branches of the arteries supplying the brain, vessel recanalization is the major factor associated with good clinical outcomes and less infarct growth. Although factors such as the quality of collateral flow also influence outcomes (1–3), recanalization is regarded as being the most important predictor of good outcomes in these patients (4, 5).

However, depending on how they are defined, large vessel occlusions occur in under a third of all patients with acute ischemic stroke (6–8). In the remaining patients, factors besides vessel recanalization potentially play a larger role in determining stroke outcome. These include the quality of collateral flow, which is important for maintaining perfusion in critically hypoperfused brain tissue (9). Tissue reperfusion, which often but not inevitably follows vessel recanalization (10–13), also affects stroke outcome beyond the effect of recanalization (12, 14). Reperfusion itself is influenced by the patency of the vessel feeding the affected area, the cerebral perfusion pressure, and the blood's flow properties (14).

It is clear that these interrelated phenomena—collateral flow, vessel recanalization, and tissue reperfusion—are essential factors contributing to the pathophysiology and progression (and thus outcome) of stroke. However, in clinical practice, these phenomena are often not measured directly. Instead, they are operationalized using measurements that are as non-invasive and convenient as possible. What is unclear is how these operationalized measurements are related to stroke outcome, as evidence regarding this has thus far been mixed (10, 15, 16).

The aim of this study was thus to investigate the relationship between MRI-based operationalized measurements of collateral flow, tissue reperfusion, and vessel recanalization on clinical and imaging outcomes. Importantly, this study aims to determine how these phenomena interact in a population where this interaction is likely most relevant—acute ischemic stroke patients with a wide range of vessel pathologies and infarct distributions.

## Materials and Methods

### Patients

This study is a retrospective analysis of 184 patients enrolled into the prospective observational 1000Plus study (clinicaltrials.org NCT00715533) at the Charité-Universitätsmedizin Berlin from September 2008 to June 2013. Inclusion criteria were MR-angiographic proven vessel occlusion on day of admission, MR imaging within 24 h after stroke onset (baseline) and perfusion imaging (PI) 24 h later (follow-up). The study design was approved by the institutional review board of the Charité-Universitätsmedizin, Berlin (EA4/026/08). Written informed consent was obtained from all patients.

### Imaging

MRI examinations were performed on a three Tesla MRI scanner (Tim Trio; Siemens AG, Erlangen, Germany) with a standard stroke imaging protocol described in detail (17).

Perfusion imaging (PI) data was analyzed using block-circulant singular value decomposition deconvolution and an arterial input function from the contralateral middle cerebral artery (18) (Stroketool version 2.8, ^©^Digital Image Solutions-HJ Wittsack).

Reperfusion was defined as the change in T_max_ > 6 s volume between baseline and follow-up as a percentage of the baseline Tmax volume. Collateral flow at baseline was assessed using the Higashida score (19) using subtracted dynamic MR perfusion source images (sMRP-SI) (20). The Higashida scale is a modification of the American Society of Interventional and Therapeutic Neuroradiology/Society of Radiology (ASITN/SIR) Collateral Flow Grading System and uses five grades to describe collateral flow of the ischemic region (19). Higashida scores were dichotomized into good (3, 4) and poor (0–2) (21). Two experienced radiologists (IG, KV, consensus reading) performed the scoring.

Perfusion volume was obtained using an in-house automated, user-independent delineation algorithm (22). From these volumes, the hypoperfusion intensity ratio (HIR) was also calculated for each patient as a different DSC-based measure of collateral flow (23). HIR was defined as the ratio between the T_max_ > 8 s volume and T_max_ > 2 s volume, as originally described (16, 23).

Vessel recanalization was classified according to the TIMI score (24) and binarized into present (TIMI = 2 or 3) or absent (TIMI = 0 or 1), as applied in previous studies (4, 12, 22, 25). Infarct growth was defined as the difference in baseline diffusion weighted imaging and fluid attenuated inversion recovery lesion volume 5 days following stroke onset.

### Statistical Analysis

Statistical analysis was performed using R Studio and R packages “Imrob” from “robustbase” package (20), and robust GLM (26). We performed a sample size calculation and found that the minimum sample size needed to detect a minimum effect size (Cohen's f^2^) of 0.1 (27) at a significance level of 0.05 and a power of 0.8 with 8 degrees of freedom (including main effects and interactions) was 158 [R package pwr (28)].

A robust generalized linear model was used to investigate the association between different clinical and imaging variables and reperfusion, long-term clinical outcome (modified Rankin Scale = mRS at day 90), and infarct growth. To account for potential differences in the factors associated with reperfusion, clinical outcome, and imaging outcome between patients with strokes in different locations, we included stroke location (anterior vs. posterior circulation) as a covariate in all models.

An alpha level *p* < 0.05 was considered as significant.

## Results

The data and analysis scripts used in this study are available at https://www.github.com/ahmedaak/1000plus_collaterals_perfusion.

We included 184 patients into our study. Patient characteristics are given in Table 1.

**Table 1:** Patient Characteristics.

**Variable**	**Value**
Age (years)	74 (65.8–82)
Sex
Male	57.6%
Female	42.4%
Time onset-to-imaging (hours)	3.4 (1.5–12)
IV thrombolysis
Yes	43.5%
No	56.5%
Circulation	
Anterior	76.6%
Posterior	23.4%
TIMI score	
0	38.6%
1	8.2%
2	23.4%
3	29.9%
NIHSS on admission	6 (3–13)
NIHSS at discharge^†^	2 (0–6)
mRS at day 90^‡^	2 (1–4)
DWI volume at baseline (mL)	5.4 (1.0–17.8)
DWI volume at follow-up (mL)	12.4 (3.0–39.1)
FLAIR volume at day 5 (mL)^§^	17.9 (5.9–54.8)
T_max_ >6 s volume at baseline (mL)	27.0 (11.0–67.2)
T_max_ >6 s volume at follow-up (mL)	7.0 (1.0–27.6)
Higashida score at baseline
0	2.2%
1	16.8%
2	26.6%
3	26.6%
4	27.2%
Higashida score at follow-up
0	1.6%
1	12.0%
2	20.7%
3	20.7%
4	43.5%
HIR at baseline	0.2 (0.1–0.4)
HIR at follow-up	0.1 (0.01–0.3)

*All values are shown as median and interquartile range (IQR) except for categorical variables, which are shown as percentages. Data is shown for the full sample of 184 patients, except where data was missing (^†^N = 183, ^‡^N = 148, ^§^N = 140). NIHSS, National Institutes of Health Stroke Scale; mRS, modified Rankin Scale; DWI, diffusion weighted imaging; FLAIR, fluid attenuated inversion recovery; TIMI score, thrombolysis in myocardial infarction score; HIR, hypoperfusion intensity ratio*.

Eighty patients (43.5%) received intravenous tissue plasminogen activator. None received mechanical thrombectomy. The distribution of vessel occlusions in the cohort is shown on Supplementary Figure 1. Eighty-six patients (46.7%) showed persistent vessel occlusion on follow-up measurements, while ninety-eight (53.3%) showed recanalization.

We ran several multiple linear regression models to test the association between reperfusion and various clinical and imaging variables. The results of the models incorporating Higashida score as a measure of collateral flow are reported here and in Supplementary Tables 1–3. The results of the models incorporating HIR and reperfusion are only shown in the Supplementary Material.

**Figure 1:**
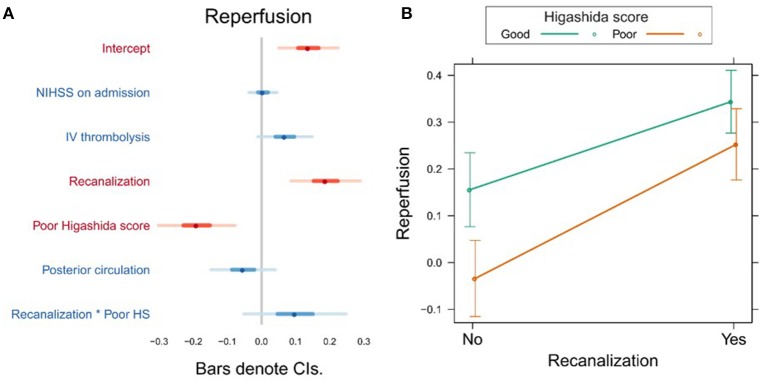
**(A)** Association between several variables and reperfusion. The dots indicate the beta coefficients derived from a robust multiple linear regression. Bars indicate the 95% confidence intervals (CI) of the estimates. Independent variables shown in red have statistically significant coefficients (*p* < 0.05). **(B)** Plot showing the predicted reperfusion values for recanalizers and non-recanalizers with good and poor Higashida scores. The plot shows that vessel recanalization and good collateral flow are associated with more reperfusion. The association between recanalization and reperfusion is similar across patients, whether or not they have good collateral flow.

In the first model, the Higashida score and recanalization were significant predictors of reperfusion (Figure 1A, Supplementary Table 1). To investigate whether the effect of recanalization on reperfusion was different depending on collateral flow quality, we included the interaction between Higashida score with recanalization status in the models. There was no significant interaction between these variables (Figure 1B, Supplementary Table 1).

### Clinical Outcome

**Figure 2:**
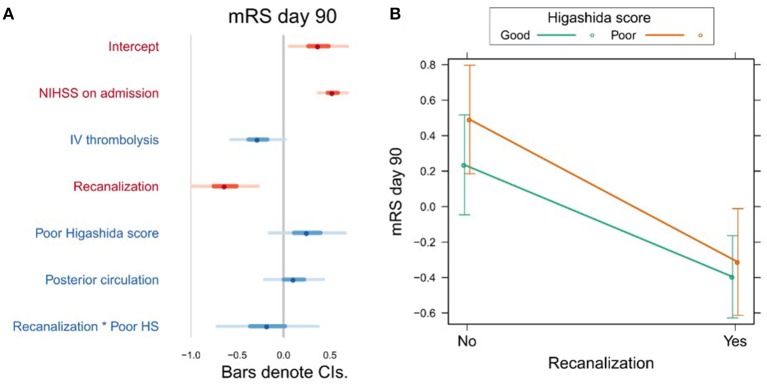
**(A)** Association between several variables and clinical outcome (mRS day 90). The dots indicate the beta coefficients derived from a robust multiple linear regression. Bars indicate the 95% confidence intervals (CI) of the estimates. Independent variables shown in red have statistically significant coefficients (*p* < 0.05). **(B)** Plot showing the predicted mRS day 90 values for recanalizers and non-recanalizers with good and poor Higashida scores. The plot shows that vessel recanalization is associated with lower mRS at day 90, but there is no significant association between Higashida score and mRS at day 90. In addition, the association between recanalization and mRS at day 90 is similar across patients, whether or not they have good collateral flow.

Variables associated with clinical outcome are shown in Supplementary Table 2. In a univariate analysis, we found a significant association between mRS day 90 and Higashida score (*b* = 0.53, *t* = 3.06, *p* = 0.003), but not with reperfusion (*b* = −0.001, *t* = −1.55, *p* = 0.124). However, neither Higashida score (*b* = 0.26, *t* = 1.21, *p* = 0.228) nor reperfusion (*b* = −0.017, *t* = −0.14, *p* = 0.891) was independently associated with mRS day 90, when NIHSS on admission, iv-tPA, recanalization, and anterior vs. posterior circulation were accounted for (Figure 2A, Supplementary Table 2). There were no significant interactions between collateral flow and recanalization on mRS day 90 (Figure 2B, Supplementary Table 2).

### Infarct Growth

**Figure 3:**
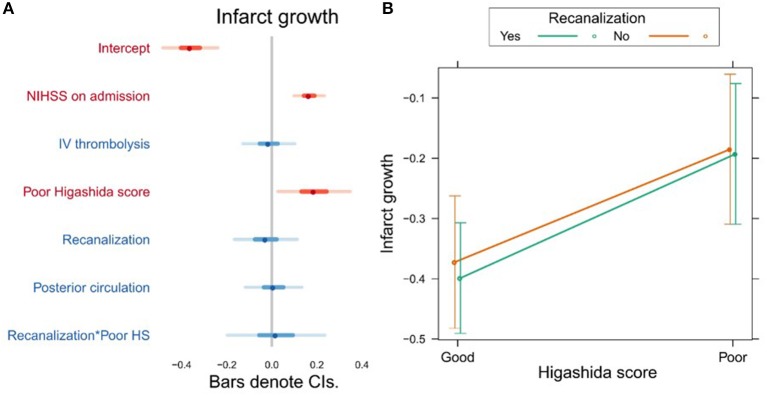
**(A)** Association between several variables and infarct growth. The dots indicate the beta coefficients derived from a robust multiple linear regression. Bars indicate the 95% confidence intervals (CI) of the estimates. Independent variables shown in red have statistically significant coefficients (*p* < 0.05). **(B)** Plot showing the predicted infarct growth values for recanalizers and non-recanalizers with good and poor Higashida scores. The plot shows that good collateral flow is associated with less infarct growth, but there is no significant association between recanalization and infarct growth. In addition, the association between collateral flow and infarct growth is similar across patients, whether or not they recanalized.

Higashida score (*b* = 0.19, *t* = 2.28, *p* = 0.025), as well as stroke severity (NIHSS) on admission (*b* = 0.17, *t* = 4.79, *p* = 0.0001), was associated with infarct growth (Figure 3A, Supplementary Table 3). There were no significant interactions between collateral flow and recanalization on infarct growth (Figure 3B, Supplementary Table 3).

## Discussion

In stroke, imaging plays an important role in selecting patients most likely to benefit from specific treatments, extending the use of established treatments to previously excluded subpopulations, and monitoring the effects of novel treatments (29). In this study, we investigated the association between several MRI-derived markers and stroke outcome. The results of this study potentially help us understand the value of these operationalized measurements in a cohort of stroke patients that is representative of patients encountered in a clinical setting.

We found that both good collateral flow (assessed using subtracted dynamic MR perfusion source images) and recanalization were independently associated with reperfusion. However, the effect of recanalization on reperfusion was similar in both patients with and without good collateral flow. Thus, far, few studies have investigated the interaction between MR-based measures of reperfusion, recanalization, and collateral flow. Studying 33 patients within 6 h of stroke onset, Makris et al. found that patients without vessel recanalization but with good collateral flow (Higashida score 3–4) had higher degrees of reperfusion than patients with poor collaterals (10). Our study confirms this finding (Figure 1B), despite the fact that our study design differed from that of Makris et al., with the first examination taking place within 24 h after stroke onset and follow-up measurements 24 h later. Our patient sample was also larger and more heterogeneous (including posterior circulation strokes, as well as proximal and distal occlusions) and our analysis accounted for potential confounding variables such as NIHSS on admission and treatment with intravenous thrombolysis. Extending the findings of Makris et al., we show that the observation that good collateral flow is associated with more reperfusion also applies to patients with vessel recanalization (Figure 1B).

Poor collateral flow was also independently associated with more infarct growth, and this relationship was similar in patients with and without vessel recanalization. The former finding is in agreement with studies using CTA (30), DSC-derived cerebral blood volume (31), the Higashida score (32) and DSA (33, 34). However, unlike previous studies (33), we did not find that patients with poor collaterals and recanalization had less infarct growth than those with poor collaterals and persistent vessel occlusion. Recanalization, on the other hand, was not independently associated with infarct growth in our study, unlike in previous studies (32, 33). A possible explanation for this could be that recanalization was assessed relatively late in our study—patients could have spontaneously recanalized any time between the baseline and follow-up exams, a period of up to 24 h. In late recanalizers, most of the infarct growth could have already occurred by the time recanalization was assessed in our study. This may also explain the discrepancy between our results and those of previous studies where recanalization was assessed immediately after endovascular therapy (32, 33). In a similar study of 44 patients that assessed recanalization >24 h after baseline MRI, it was also found that recanalization was not independently associated with infarct growth (34).

Our study showed that neither reperfusion nor collateral flow at baseline was independently associated with long-term clinical outcome. Studies on whether collateral flow predicts clinical outcome in relation to recanalization have yielded contradictory findings. In 60 patients, Marks et al. (33) found no difference in clinical outcome between patients with poor and good collateral flow (assessed using digital subtraction angiography) who recanalized. On the other hand, in the largest MR perfusion-based study to date using the Higashida score (*n* = 134), Kim et al. found that both good collateral flow and recanalization were independently associated with good clinical outcome (15). In agreement with the results of Marks et al. (33), we found that recanalization was independently associated with good clinical outcomes, and this effect did not significantly differ between patients with different collateral flow grades (Figure 2B). The lack of association between collateral flow and clinical outcome, which runs contrary to the findings of Kim et al. (15), may be due to the differences in the studied stroke population. Whereas, Kim et al. (15) exclusively studied patients with proximal (M1/ICA) vessel occlusions eligible for thrombectomy, our sample was more diverse. The utility of the Higashida score of collateral flow in such a diverse sample of stroke patients should be further investigated in future studies.

Interestingly, several lines of evidence suggest that the potential effect of collateral flow on clinical outcome may be more complicated than previously thought. Firstly, collateral flow is dynamic and changes over time. Yeo et al. (35) found that an improvement in collateral grade over time assessed using CTA in patients without recanalization was associated with worse outcome and mortality. Such slower recruitment of collaterals is mediated by metabolic factors and angiogenesis, as compared to the rapid response due to drop in perfusion pressure and relaxation of smooth muscles with a resulting pressure gradient (36). The detrimental effect of this delayed response might be due to maximal vasodilation of collaterals in the ischemic area and a steal-like effect by recruitment of collaterals in adjacent regions resulting in infarct progression. Another explanation could be failed autoregulation in the affected area with reperfusion injury. Campbell et al. (16) also pointed out that fluctuations in collateral flow over time underscore the problem of accurately predicting infarct growth based on perfusion lesions derived from single thresholds. Depending on individual factors, these changes in collateral flow over time determine which regions of hypoperfused tissue progress to irreversible damage.

Another issue that complicates collateral flow assessment is methodological. Perfusion measurements using either MRI or CT cannot differentiate between rapid collaterals successfully maintaining perfusion to preserve and save brain tissue or collateral flow to increase regional cerebral perfusion above normal, which might in turn be harmful (37). This distinction is important, because the temporal behavior of collateral filling seems to be relevant for infarct evolution. Beyer et al. (38) demonstrated that faster collateral filling (assessed using dynamic CTA reconstructed from whole-brain CT perfusion raw data) was associated with smaller follow-up infarct lesions, an effect which was independent of extent of collateralization.

The limitations of our study include its single-center observational nature and the fact that recanalization and reperfusion were assessed 24 h following the initial MR examination. It is also important to note that overall, our study was powered to detect small-to-medium effect sizes (Cohen's f^2^ = 0.1) (31), and it is therefore possible that very small effects could have been missed. This is particularly likely in the analyses of long-term clinical outcome and infarct growth, where incomplete patient data reduced the analyzed sample size (to 149 and 141, respectively). On the other hand, our study is one of the largest analyses thus far of the influence of MRI-based imaging variables on clinical and imaging stroke outcomes. Although the cohort we studied here is heterogeneous, it possibly reflects better the kind of stroke population encountered in clinical routine. Most previous MR-based studies (10, 15, 39–41) have restricted the assessment of reperfusion, collateral flow, and recanalization to patients with occlusions of proximal vessels of the anterior circulation, as these have thus far been the main target for mechanical thrombectomy. However, endovascular therapy is increasingly being performed in more distal vessel occlusions as well as posterior circulation occlusions, and imaging-based biomarkers of outcome are also potentially relevant for patients who do not undergo thrombectomy. It is therefore important for such biomarkers to be investigated in wider patient cohorts, and our study presents a first step in doing so.

## Conclusion

MR-based measures of collateral flow and vessel recanalization are independently associated with reperfusion and infarct growth. The association between good collateral flow and more reperfusion or less infarct growth holds true regardless of recanalization status. Clinical outcome, on the other hand, is associated with baseline stroke severity and recanalization, but not DSC-MRI-based collateral flow status in this cohort of stroke patients with a range of vessel occlusion and stroke locations.

## Data Availability Statement

The datasets generated for this study are available on request to the corresponding author.

## Ethics Statement

The studies involving human participants were reviewed and approved by Institutional review board of the Charité-Universitätsmedizin, Berlin (EA4/026/08). The patients/participants provided their written informed consent to participate in this study.

## Author Contributions

KV contributed to concept and design of this study, participated in data acquisition, analysis, interpretation of data, and drafting the manuscript. SZ contributed to data acquisition and analysis. IG contributed to data acquisition, analysis, interpretation of data, and participated in drafting the manuscript. CN contributed to interpretation of data and drafting the manuscript. JF contributed to concept and design of the study and participated in drafting the manuscript. AK performed the statistical analysis, participated in interpretation of data, and contributed in drafting the manuscript.

### Conflict of Interest

KV and JF were funded by the German Federal Ministry of Education and Research (01EO0801, 01EO01301). JF has received consulting, lecture, advisory board fees from BioClinica, Cerevast, Artemida, Brainomix. CN has received consulting, lecture fees from Boehringer Ingelheim, W. L. Gore and Associates, Bristol-Myers Squibb, Pfizer, Sanofi. AK, KV, and JF were co-inventors of a patent application relating to a method for automated, user-independent delineation of perfusion lesions, used in this manuscript. The remaining authors declare that the research was conducted in the absence of any commercial or financial relationships that could be construed as a potential conflict of interest.
