# Supplementary material for: The Association Between Recanalization, Collateral Flow, and Reperfusion in Acute Stroke Patients: A Dynamic Susceptibility Contrast MRI Study
Source: Front Neurol. 2019 Oct 25;10:1147. doi: 10.3389/fneur.2019.01147 (PMC6823193; doi:10.3389/fneur.2019.01147)
Supplement: Supplementary file 1 [file Table_1.DOCX]

**SUPPLEMENTAL MATERIAL**

**Supplementary table 1** Predictors of reperfusion

|  | | |
| --- | --- | --- |
|  | **HIR model** | **Higashida model** |
| NIHSS on admission | -0.011 (0.024) | 0.005 (0.021) |
|  | t = -0.440 | t = 0.213 |
|  | p = 0.661 | p = 0.832 |
| IV thrombolysis | 0.059 (0.043) | 0.069^*^ (0.041) |
|  | t = 1.372 | t = 1.687 |
|  | p = 0.172 | p = 0.094 |
| Recanalization | **0.242^***^ (0.042)** | **0.188^***^ (0.052)** |
|  | **t = 5.774** | **t = 3.614** |
|  | **p = 0.00000** | **p = 0.0004** |
| HIR | -0.033 (0.032) |  |
|  | t = -1.008 |  |
|  | p = 0.315 |  |
| Higashida score (poor) |  | **-0.190^***^ (0.058)** |
|  |  | **t = -3.294** |
|  |  | **p = 0.002** |
| Circulation (posterior) | -0.071 (0.052) | -0.054 (0.048) |
|  | t = -1.367 | t = -1.111 |
|  | p = 0.174 | p = 0.268 |
| Recanalization*HIR | 0.041 (0.042) |  |
|  | t = 0.970 |  |
|  | p = 0.334 |  |
| Recanalization*Higashida (poor) |  | 0.099 (0.077) |
|  |  | t = 1.288 |
|  |  | p = 0.200 |
|  | | |
| Observations | 178 | 178 |
| R^2^ | 0.226 | 0.283 |
| Adjusted R^2^ | 0.199 | 0.257 |
| Residual Std. Error (df = 171) | 0.260 | 0.246 |
|  | | |

NIHSS=National Institutes of Health Stroke Scale, HIR=hypoperfusion intensity ratio. *p<0.1; **p<0.05; ***p<0.01

**Supplementary table 2** Predictors of long-term clinical outcome (mRS day 90)

|  | **HIR model** | **Higashida model** | **Reperfusion model** |
| --- | --- | --- | --- |
| NIHSS on admission | **0.511^***^ (0.084)** | **0.531^***^ (0.081)** | **0.557^***^ (0.080)** |
|  | **t = 6.116** | **t = 6.554** | **t = 6.999** |
|  | **p = 0.000** | **p = 0.000** | **p = 0.000** |
| IV thrombolysis | -0.281^*^ (0.149) | -0.276^*^ (0.151) | **-0.312^**^ (0.151)** |
|  | t = -1.886 | t = -1.835 | **t = -2.062** |
|  | p = 0.062 | p = 0.069 | **p = 0.042** |
| Recanalization | **-0.740^***^ (0.145)** | **-0.631^***^ (0.183)** | **-0.663^***^ (0.179)** |
|  | **t = -5.113** | **t = -3.449** | **t = -3.711** |
|  | **p = 0.00001** | **p = 0.001** | **p = 0.0003** |
| HIR | 0.107 (0.109) |  |  |
|  | t = 0.979 |  |  |
|  | p = 0.330 |  |  |
| Higashida (poor) |  | 0.256 (0.211) |  |
|  |  | t = 1.213 |  |
|  |  | p = 0.228 |  |
| Reperfusion |  |  | -0.017 (0.121) |
|  |  |  | t = -0.138 |
|  |  |  | p = 0.891 |
| Circulation (posterior) | 0.102 (0.163) | 0.112 (0.162) | 0.116 (0.166) |
|  | t = 0.626 | t = 0.690 | t = 0.698 |
|  | p = 0.533 | p = 0.492 | p = 0.487 |
| Recanalization*HIR | 0.030 (0.144) |  |  |
|  | t = 0.209 |  |  |
|  | p = 0.835 |  |  |
| Recanalization*Higashida (poor) |  | -0.173 (0.279) |  |
|  |  | t = -0.619 |  |
|  |  | p = 0.538 |  |
| Recanalization*Reperfusion |  |  | -0.279 (0.390) |
|  |  |  | t = -0.716 |
|  |  |  | p = 0.476 |
|  | | | |
| Observations | 148 | 148 | 143 |
| R^2^ | 0.396 | 0.390 | 0.388 |
| Adjusted R^2^ | 0.371 | 0.364 | 0.361 |
| Residual Std. Error | 0.804 (df = 141) | 0.808 (df = 141) | 0.810 (df = 136) |

NIHSS=National Institutes of Health Stroke Scale, HIR=hypoperfusion intensity ratio. *p<0.1; **p<0.05; ***p<0.01

**Supplementary Table 3** Predictors of total infarct growth

|  | | | |
| --- | --- | --- | --- |
|  | **HIR model** | **Higashida model** | **Reperfusion model** |
| NIHSS on admission | **0.157^***^ (0.037)** | **0.166^***^ (0.035)** | **0.184^***^ (0.037)** |
|  | **t = 4.194** | **t = 4.792** | **t = 4.984** |
|  | **p = 0.00005** | **p = 0.00001** | **p = 0.00001** |
| IV thrombolysis | **-0.005 (0.061)** | **-0.014 (0.059)** | **0.003 (0.063)** |
|  | t = -0.080 | t = -0.233 | t = 0.047 |
|  | p = 0.936 | p = 0.817 | p = 0.963 |
| Recanalization (yes) | -0.055 (0.061) | -0.027 (0.071) | -0.024 (0.076) |
|  | t = -0.914 | t = -0.372 | t = -0.312 |
|  | p = 0.363 | p = 0.711 | p = 0.756 |
| HIR | **0.103^**^ (0.048)** |  |  |
|  | **t = 2.120** |  |  |
|  | **p = 0.036** |  |  |
| Higashida (poor) |  | **0.187^**^ (0.082)** |  |
|  |  | **t = 2.279** |  |
|  |  | **p = 0.025** |  |
| Reperfusion |  |  | 0.016 (0.050) |
|  |  |  | t = 0.327 |
|  |  |  | p = 0.745 |
| Circulation (posterior) | 0.007 (0.066) | 0.008 (0.064) | 0.002 (0.070) |
|  | t = 0.106 | t = 0.125 | t = 0.027 |
|  | p = 0.916 | p = 0.901 | p = 0.979 |
| Recanalization*HIR | -0.007 (0.063) |  |  |
|  | t = -0.118 |  |  |
|  | p = 0.907 |  |  |
| Recanalization*Higashida (poor) |  | 0.019 (0.110) |  |
|  |  | t = 0.171 |  |
|  |  | p = 0.865 |  |
| Recanalization*Reperfusion |  |  | -0.048 (0.157) |
|  |  |  | t = -0.305 |
|  |  |  | p = 0.762 |
|  | | | |
| Observations | 140 | 140 | 137 |
| R^2^ | 0.248 | 0.261 | 0.185 |
| Adjusted R^2^ | 0.215 | 0.228 | 0.147 |
| Residual Std. Error | 0.321 (df = 133) | 0.312 (df = 133) | 0.336 (df = 130) |

NIHSS=National Institutes of Health Stroke Scale, HIR=hypoperfusion intensity ratio. *p<0.1; **p<0.05; ***p<0.01

**Supplementary Figure 1**


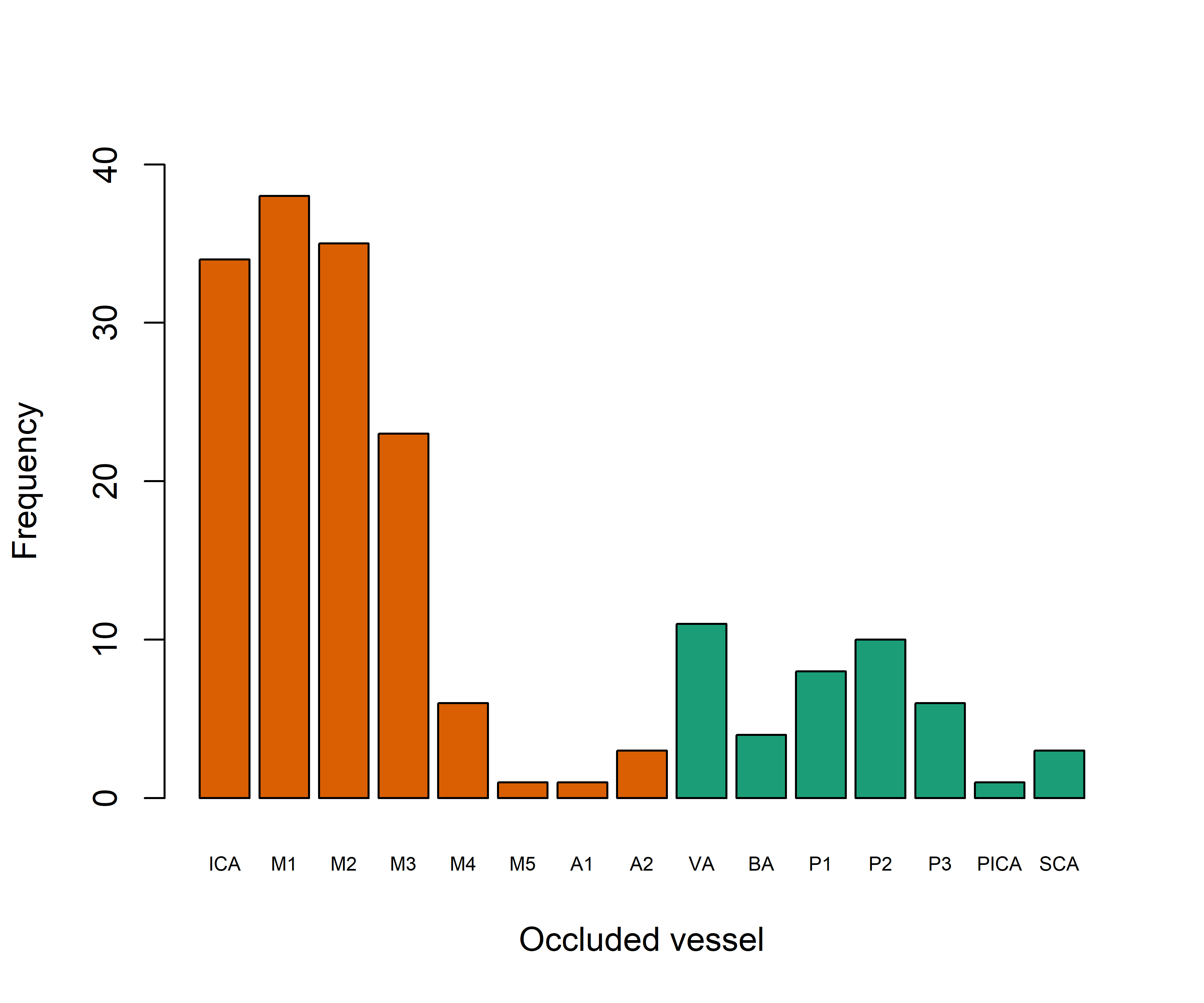


*The distribution of vessel occlusions in the cohort for vessels of the anterior (orange) and posterior (green) circulation. Frequency refers to number of patients. ICA; internal carotid artery, M; branches of the middle cerebral artery, A; branches of the anterior cerebral artery, VA; vertebral artery, BA; basilar artery, P; branches of the posterior cerebral artery, PICA; posterior inferior cerebellar artery, SCA; superior cerebellar artery.*
